# Recurrent Hyponatremia in an Elderly Patient With a Cystic Pituitary Gland

**DOI:** 10.5812/numonthly.3931

**Published:** 2012-06-20

**Authors:** Pavan Malleshappa, Ravi Ranganath, Anup P. Chaudhari, Preeti Singhai, Bharat V. Shah

**Affiliations:** 1 Nephrology Department, Vaatsalya Hospital, Bharathi Healthcare Complex, R C Road, Hassan, Karnataka State, India; 2 Department of Nephrology, Lilavati Hospital and Research Centre, Mumbai, India

**Keywords:** Cerebral Salt Wasting, Hyponatremia, Pituitary Gland, Cyst

## Abstract

A 71-year-old male with a long history of diabetes and hypertension was admitted with mild azotemia and recurrent hyponatremia. He was diagnosed with a pituitary gland cystic tumor. On careful evaluation, his hyponatremia was found to be due to cerebral salt wasting. The patient made a full recovery following treatment for cerebral salt wasting.

## 1. Introduction

Hyponatremia is a common disorder resulting from a wide variety of causes. Treatment can vary from fluid restriction to rehydration, depending on the pathogenesis of the disorder. Perhaps, more importantly for individual cases, the presence of hyponatremia in patients with pituitary gland cystic tumors often leads to a premature diagnosis of SIADH without careful elimination of other potentially correctable causes of hyponatremia (such as solute depletion, hypothyroidism, adrenal insufficiency, and cerebral salt wasting (CSW)), sometimes resulting in inappropriate or ineffective therapies. Careful evaluation is necessary to determine pathogenesis of hyponatremic disorders.

## 2. Case Report

A 71-year-old male with a 30-year history of diabetes mellitus and hypertension and osteoarthritis of both knee joints was in good health until a month before admission to our hospital, at which time he developed anorexia, nausea, and generalized weakness. When his condition further worsened and he began to experience drowsiness, he was admitted to another hospital. Studies at this hospital included routine urinalysis, which showed 1^+^ protein (the rest were unremarkable); CBC showed an Hb level of 11.4 g/dL, a WBC count of 6800/cmm, and a platelet count of 351,000/cmm. Serum creatinine was 1.96 mg/dL, sodium was 116 meq/L, potassium was 4.4 meq/L, and spot urinary sodium was 88 meq/L. Ultrasound of the abdomen and pelvis was unremarkable and brain MRI was normal.

The patient was diagnosed with diabetic nephropathy and hyponatremia due to SIADH and treated with intravenous hypertonic saline. His serum sodium improved to 128 meq/L over the next 3 days. With this treatment, his clinical condition improved and he was discharged. He was readmitted 2 more times at the same hospital with similar symptoms and hyponatremia. Each time he improved with the administration of hypertonic saline.

The patient was brought to our hospital because of his recurrent episodes of hyponatremia. On admission, he was not in acute distress but appeared to be mildly dehydrated. His pulse was 88/min and his blood pressure was 140/80 mm Hg while lying down and 110/70 mm Hg while standing. Studies on admission revealed normal urinalysis, CBC, blood sugar levels, and liver function tests. The rest of the studies are presented in Table 1.

**Table 1:** Laboratory Investigations

	**Amounts**
BUN ^a^, mg/dL	12
Serum Creatinine, mg/dL	1.59
Serum Sodium, meq/L	128
Serum Potassium, meq/L	4
Serum Chloride, meq/L	90
Serum Bicarbonate, meq/L	18.6
Serum Uric acid, mg/dL	9.45
Urine Sodium, meq/L	104
Urine Osmolality, mOsm/kg	260

^a^Abbreviation: BUN; blood urine nitrogen

Since he appeared to be volume depleted, the patient was treated with a normal saline infusion. After this treatment, he started passing large amounts of urine (~5 L/day). His serum sodium progressively improved, and consequently, his creatinine also normalized. He was discharged with normal serum sodium and creatinine levels. A month after his discharge, he was reevaluated and found to have normal sodium and creatinine levels. Additional studies performed as part of the evaluation of hyponatremia included thyroid and adrenal function (Table 2). In view of his low T3, T4, TSH, ACTH, and cortisol levels, we considered the possibility of a pituitary problem.

**Table 2:** Pituitary Function Tests

	**Observed Value**	**Normal Range**
T3, ng/mL	0.66	0.79-1.49
T4, mcg/dL	1.44	4.5-12
TSH ^a^, μIU/mL	0.44	0.49-4.67
Serum Cortisol; 8am, mcg/dL	0.2	6.2-19.4
Serum Cortisol; 4pm, mcg/dL	0.14	2.3-11.9
ACTH ^a^, pg/mL	7	7.2-63.3

^a^Abbreviations: ACTH; adrenocorticotropic hormone, TSH; thyroid-stimulating hormone

**Figure 1:**
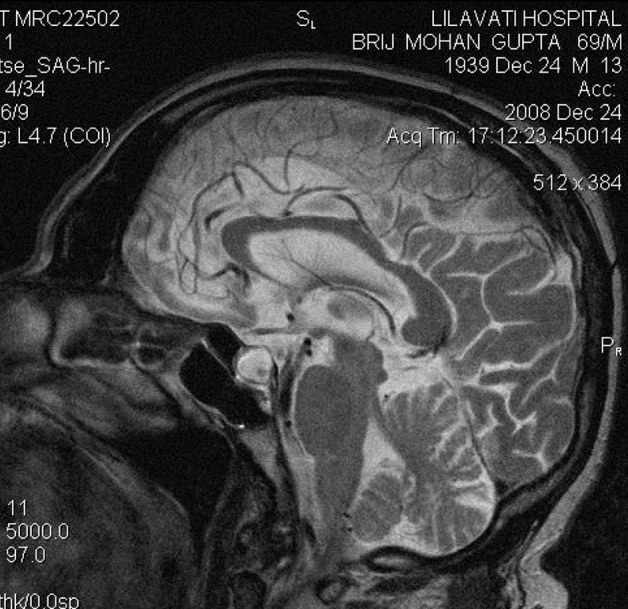
T2W Saggital Section Showing Cystic Pituitary Gland

**Figure 2:**
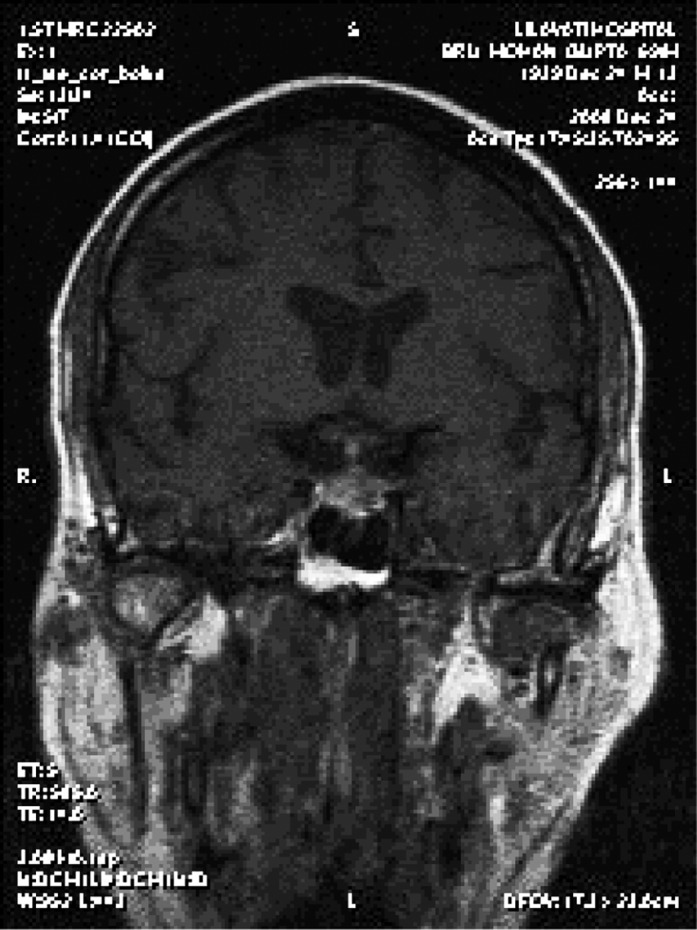
Contrast Enhanced Predominantly Cystic Pituitary Gland

Although the brain MRI performed externally was reported as normal, a repeat MRI with special pituitary cuts was performed. This showed an enlarged and predominantly cystic pituitary gland (Figure 1 and Figure 2). He was treated with thyroxine and prednisolone, and his clinical condition improved completely.

## 3. Discussion

There are three issues to be discussed in this case. First, during the initial admission of this patient to a different hospital, azotemia was diagnosed as diabetic nephropathy. However, the azotemia was actually due to a prerenal state, because it rapidly resolved with saline infusion. Making this differentiation between the two conditions has an important practical implication. Diabetic nephropathy would signify that this patient would experience a progressive downhill course with the development of end-stage kidney disease. On the other hand, a prerenal state would completely reverse with hydration, as was observed in this case.

Second, hyponatremia was initially attributed to SIADH. While high urinary sodium along with hyponatremia is a feature of SIADH, other features were not consistent with SIADH. The patient was actually volume depleted, a feature not consistent with SIADH. Moreover, the diagnosis of SIADH would require ruling out thyroid and adrenal dysfunction, which was not done in this case. Our patient had hypothyroidism and cortisol deficiency. The combination of volume depletion, hypothyroidism, and cortisol deficiency may have caused elevated ADH levels, which were suppressed with volume repletion, as suggested by diuresis that increased with correction of volume.

An important question is “What caused volume depletion in this patient? Could it be due to hypoaldosteronism?” Serum aldosterone levels were not measured in this case, but hypoaldosteronism was unlikely because ACTH acts only on glucocorticoids and not on mineralocorticoids. Moreover, serum potassium levels were normal in this case. We would have expected hyperkalemia with hypoaldosteronism; however, hyperkalemia is absent in ~50% of patients with primary adrenal insufficiency, which may also be a possibility in the present case.

CSW could have caused volume depletion (1). Patients with CSW are characterized by high urine output at the time of diagnosis and a further increase in urine output after volume repletion due to the removal of the hypovolemic stimulus for ADH release. Excretion of dilute urine leads to correction of hyponatremia. Indeed, this was observed in our patient. He had high urine output despite being volume depleted on admission and had massive diuresis with correction of hyponatremia after volume repletion. It could be argued that hyponatremia in our patient may have been due to hormonal deficiency. Hypothyroidism and hypoadrenalism lead to hyponatremia in euvolemic states. This patient was in a state of gross dehydration with good urine output at the time of presentation, and his hyponatremia improved following administration of intravenous fluids and not with hormonal supplementation.

The third issue in this case is that although this patient had a pituitary mass, the mass was overlooked in the MRI performed at the other hospital. This indicates that special cuts need to be taken to visualize pituitary tumors. MRI of the pituitary region, involving fine cuts and sagittal and coronal reconstruction, is the gold standard imaging method for pituitary disease (2). In summary, this case highlights three important conclusions. First, it is important to identify non-diabetic kidney disease in diabetic patients with azotemia because the prognosis may be significantly different. Second, SIADH and CSW are two potential causes of hyponatremia in patients with neurological disorders. The primary distinction lies in the assessment of the volume status (3). SIADH is a volume-expanded state due to ADH-mediated renal water retention. CSW is characterized by a volume-contracted state resulting from renal salt wasting. Making an accurate diagnosis is important because the therapy for each condition is quite different; vigorous salt replacement is indicated in patients with CSW, while fluid restriction is the treatment of choice in patients with SIADH. Third, proper imaging is important for visualizing pituitary tumors.
